# Inferences about fossil hominin locomotion through 3D morphometric analysis of wrist ligament insertion sites

**DOI:** 10.1038/s41598-025-26487-y

**Published:** 2025-11-27

**Authors:** Aroa Casado, Xavier Martínez-Liria, Marta San-Millán, Laura Menés, Neus Ciurana, Marcel García-Cuesta, Patrícia Rodríguez, Francisco Pastor, Roberto Cabo, Josep Maria Potau

**Affiliations:** 1 Unit of Human Anatomy and Embryology, Faculty of Medicine and Health Sciences, University of Barcelona, C/Casanova 143, 08036 Barcelona, Spain; 2 Institute of Archaeology (IAUB), Faculty of Geography and History, University of Barcelona, 08001 Barcelona, Spain; 3 Department of Medical Sciences, Clinical Anatomy, Embryology and Neuroscience Research Group (NEOMA), Faculty of Medicine, University of Girona, C/Emili Grahit 77, 17071 Girona, Spain; 4 Physiotherapy Unit, Faculty of Medicine and Health Sciences, Universitat Rovira i Virgili, 43204 Reus, Spain; 5 Osteology and Comparative Anatomy Research Group, Department of Anatomy and Radiology, University of Valladolid, C/Ramón y Cajal 7, 47005 Valladolid, Spain; 6 Department of Human Anatomy and Histology, University of Salamanca, C/Alfonso X el Sabio s/n, 37007 Salamanca, Spain

**Keywords:** Wrist anatomy, Ligament insertions, Hominin evolution, Locomotor behavior, Geometric morphometrics, Fossil primates, Anatomy, Evolution, Zoology

## Abstract

Understanding the evolution of wrist anatomy in fossil hominins is essential for reconstructing their locomotor behavior and manipulative capabilities. Traditionally, most studies have focused on bone morphology, overlooking the informative potential of soft tissue attachment sites. In this study, we introduce a novel approach based on the three-dimensional geometric morphometric analysis of ligament insertion sites on the distal radial epiphysis. We analyzed a comparative sample including fossil hominins—*Australopithecus afarensis*, *Australopithecus anamensis*, *Australopithecus sediba*, *Paranthropus robustus*, *Homo neanderthalensis*, and archaic *Homo sapiens*—as well as extant hominoids: *Homo sapiens*, *Pan troglodytes*, *Gorilla gorilla* and *Pongo pygmaeus*. The results show marked interspecies differences in the size, orientation, and position of specific ligament insertions, reflecting divergent functional adaptations. Notably, the morphology of these insertions aligns with known behavioral and locomotor patterns described for these species, highlighting the reliability of ligament morphology as a proxy for inferring habitual activity in extinct taxa. This research expands the methodological toolkit available for paleoanthropology and emphasizes the relevance of soft-tissue-related structures in understanding hominin evolution beyond bone morphology alone.

## Introduction

Throughout evolutionary history, locomotor behavior has been a fundamental element in the adaptation of primates to a wide variety of environments and ecological niches. This adaptive process has led to a remarkable diversity of locomotor strategies, including brachiation, knuckle-walking, suspensory behaviors, and bipedalism, making primates an ideal model for comparative studies of the anatomy of the locomotor system^1^.

The ability of primates to adapt to different habitats and ways of life has been a key factor in their evolution and success as a taxonomic group^2^. Traditionally, the study of locomotion in humans from an evolutionary perspective has focused predominantly on the lower limb, due to its crucial importance in understanding the evolution of bipedalism, one of the most distinctive features of humans^3–5^. In order to better understand bipedalism, researchers have addressed several aspects, including bone structure and biomechanics, with methods ranging from detailed analyses of bone anatomy to simulation of movements^6–10^. Research by Ruff^11–13^ has further demonstrated that cortical bone geometry and robusticity encode stable loading patterns, allowing locomotor adaptations in species such as *Australopithecus* and *Homo erectus* to be reconstructed. These findings highlight that skeletal structures contain both phylogenetic signals and biomechanical information related to functional use, a critical framework for interpreting wrist and hand morphology.

In recent years, however, research has increasingly focused on the evolution of the upper limb, not only in terms of its role in locomotion but also in its manipulative and functional capacities. This reflects a broader interest in understanding manual behavior from both functional and evolutionary perspectives^14–16^. This emphasis has been supported by the development of quantitative methods, such as three-dimensional geometric morphometrics (3D GM), which allow for detailed characterization of ligamentous and muscular attachment sites and provide reliable insights into functional adaptations.

In this context, it is essential to recognize that evolution and plasticity are inseparable processes in entheses, including ligamentous insertions. These structures exhibit a highly conserved zonal organization—fibrous tissue, uncalcified fibrocartilage, calcified fibrocartilage, and bone—representing an evolutionary design optimized to withstand repetitive loading in joints such as the wrist^17,18^. At the same time, their microstructure and cortical bone topography dynamically respond to mechanical stimuli over an individual’s lifetime, as predicted by the mechanostat paradigm^19^ and confirmed by studies linking enthesis stiffness with bone microarchitecture^20^.

Our group has provided empirical evidence for this dual nature: in hylobatids, palmar radiocarpal ligament insertions display unique configurations associated with brachiation^21^, while captive hominoids demonstrate detectable morphological changes in ligament attachment areas in response to altered wrist use^22^. Together, these studies reinforce the view that both cortical bone and entheses act as anatomical archives that integrate phylogenetic information with biomechanical loading signals, offering essential context for interpreting morphological variation in the wrist and hand.

Historically, the interpretative value of entheseal morphology for behavioral reconstruction was questioned due to the multicausal nature of bone–soft tissue interaction and the influence of confounding variables such as age, health status, and genetic predisposition^23–25^. However, recent validation studies using controlled experiments and comparative anatomy have re-established the potential of entheses for behavioral reconstruction, especially when applying advanced tools such as landmark-based geometric morphometrics (GM) and the VERA method^26–28^. These findings have also been supported by studies focused specifically on the wrist, which demonstrated the repeatability and reliability of 3D morphometric analyses applied to ligament insertion sites on the distal radius^21,22,29^. Additionally, complementary research has documented anatomical adaptations of the upper limb associated with different locomotor or manipulative uses in hominoid primates, including comparative analyses of elbow extensors and deltoid muscle structure in chimpanzees and humans^30,31^.

Further support comes from studies on entheseal 3D shape as a correlate not only of habitual activity but also of biomechanical efficiency, particularly in relation to the force-producing capacity of the attaching musculature. This has been explored in detail using GM analyses in relation to joint torque and moment arms^32^, reinforcing the hypothesis that 3D entheseal shape may reflect genetically guided morphological optimization rather than—or in addition to—plasticity due to individual loading histories.

These findings align with other internal bone architecture analyses, such as trabecular morphology, which have shown responses to habitual mechanical loading during life^33,34^. Importantly, when combined, these approaches suggest that certain early hominins may have used their hands in ways more similar to modern humans than previously assumed, even if their external morphology remains primitive^35^.

Recent studies have also shed light on the evolution of the upper limb through comprehensive analyses of its morphology^30,31,36–39^. These studies have examined diverse anatomical features, such as bone morphology and muscle insertions, and have provided a deeper understanding of how primates have adapted their upper limbs for climbing, object manipulation, and even communication^28^.

The integration of approaches such as 3D GM, curvature-based algorithms, and biomechanical modeling is therefore essential to disentangle the multicausal nature of entheseal morphology—balancing signals of plasticity with genetically encoded form^25,40^. In light of these developments, new methodological contributions continue to expand the range of anatomical structures that can inform reconstructions of behavior and functional adaptation.

To overcome the challenge of inferring behavior from skeletal remains, our group has developed a novel methodology based on the morphometric analysis of ligament insertion sites in the distal radial epiphysis of hominoid primates^29^. Our methodology is based on the premise that morphological features of ligament insertion sites in bones can offer valuable information about the locomotor behavior of primate species. To validate this methodology, we performed a detailed analysis of wrist ligaments in several primate species^51^ and found that ligament insertion sites in the distal radial epiphysis can be used to reliably identify locomotor behavior patterns^29^.

Building on this prior validation, the present study applies this methodology to a sample of fossil hominoid primates to analyze the three-dimensional morphology of the insertion sites of the three ligaments that compose the palmar radiocarpal complex: the radioscaphocapitate (RSC), long radiolunate (LRL), and short radiolunate (SRL). We examined key representatives of several species, including *Australopithecus afarensis* (AL 288-1Q and AL 288–1 V), the paratype of *Australopithecus sediba* (MH2 88–142), *Australopithecus anamensis* (KNM-ER-20419), *Paranthropus robustus* (SK 3602), *Homo neanderthalensis* (Tabun C4 and Spy), and archaic *Homo sapiens* (Skhul 1). These species span a broad range of hominin taxa and time periods, enabling meaningful evolutionary comparisons.

The main objective of our study was to explicitly test the hypothesis that the three-dimensional geometric morphometric (3D GM) configuration of the palmar radiocarpal ligament insertions reflects functional and evolutionary adaptations associated with locomotor and manipulative patterns, allowing inferences about the habitual behavior of fossil hominin species. Specifically, we sought to assess whether the classification of the locomotion of these species in the existing literature aligns with the morphometric results obtained from the analysis of distal radial entheses. This hypothesis builds on our team’s previous research, which demonstrated that variation in the locomotor repertoire of extant great apes is closely associated with the morphology of the palmar radiocarpal ligament insertion sites. Our prediction was that variation in the shape, size, and anatomical position of these entheses would mirror the locomotor and manipulative repertoires documented in extant great apes and humans, providing a comparative framework to interpret fossil specimens. To this end, we conducted a comprehensive three-dimensional geometric morphometric (3D GM) analysis of the ligament insertions, evaluating their shape, size, and relative anatomical position. We also compared these fossil data with those of extant species (*Homo sapiens*, *Pan troglodytes*, *Gorilla gorilla*, and *Pongo pygmaeus*).

By focusing on ligamentous entheses rather than gross bone morphology, this study offers a more detailed perspective on locomotor and manipulative behavior in extinct hominoids. Moreover, it builds upon a growing body of interdisciplinary research that recognizes entheseal morphology as the product of both evolutionary functional adaptation and individual life history. This dual lens allows for a more nuanced understanding of the evolution of the primate musculoskeletal system, especially in archaeological and paleontological contexts where fossil preservation is often incomplete.

## Materials and methods

### Osteological samples

We analyzed a total of 110 radii (Table 1), 102 of which were from extant species: *Homo sapiens* (*N* = 31), *Pan troglodytes* (*N* = 25), *Gorilla gorilla* (*N* = 31) and *Pongo pygmaeus* (*N* = 15). An additional eight fossil specimens came from the following extinct species: *Australopithecus afarensis* (AL 288-1Q and AL 288–1 V), the paratype specimen of *Australopithecus sediba* (MH2 88–142), *Australopithecus anamensis* (KNM-ER-20419), *Paranthropus robustus* (SK 3602), *Homo neanderthalensis* (Tabun C4 and Spy) and archaic *Homo sapiens* (Skhul 1). The human specimens came from adult cadavers with a mean age of 80.9 years (range 38–97 years) and were provided by the Unit of Human Anatomy and Embryology of the University of Barcelona (UB, Spain). The non-human primate specimens came from wild-caught adults and were provided by the Anthropological Institute and Museum of the University of Zurich (UZH, Switzerland). The fossil specimens were provided by the Center for the Study of Human Origins (CSHO) in the Department of Anthropology at New York University (New York, NY, USA). All fossil data were acquired from first-generation casts produced directly from the original fossils using high-precision molding techniques, ensuring minimal dimensional distortion.

**Table 1:** Sources of extant and fossil primate specimens included in the study.

Specimens	*N*	Source
*Homo sapiens*	31	Unit of Human Anatomy and Embryology, University of Barcelona (UB, Spain)
*Pan troglodytes*	25	Anthropological Institute and Museum, University of Zurich (UZH, Switzerland)
*Gorilla gorilla*	31	
*Pongo pygmaeus*	15	
Fossil hominins	8	Center for the Study of Human Origins (CSHO), Department of Anthropology, New York University (NY, USA)

### 3D GM analysis

The distal radial epiphyses of the current apes and humans were digitized using a NextEngine Ultra HD laser surface scanner at a resolution of 0.1 mm point spacing, with a density of 40k (2×) points per scan. All scanning and post-processing steps followed standardized protocols to ensure high morphometric fidelity. Multiple scan sections were aligned and merged using the *Volume Merge* option in NextEngine HD software at a resolution of 0.5 mm, and the resulting meshes were saved in PLY format. The triangular meshes were refined and edited using the open-source software MeshLab^42^, and landmark acquisition was performed in Landmark Editor software (v.3.6)^43^.

**Fig. 1:**
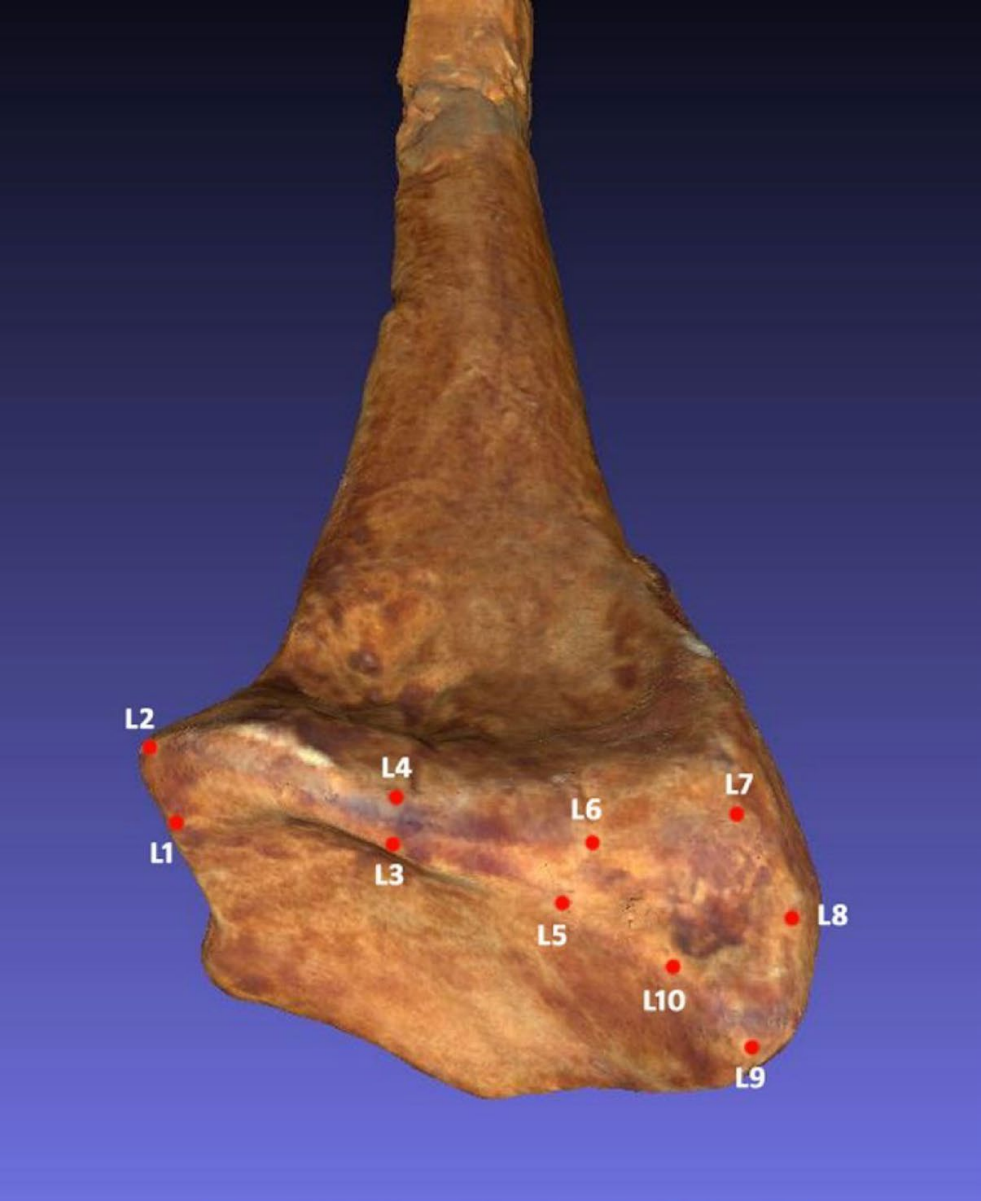
Landmarks used to delimit the ligament insertion sites in the distal epiphysis of the radius of archaic *Homo sapiens* (Skhul 1) (based on Casado et al., 2019).

**Fig. 2:**
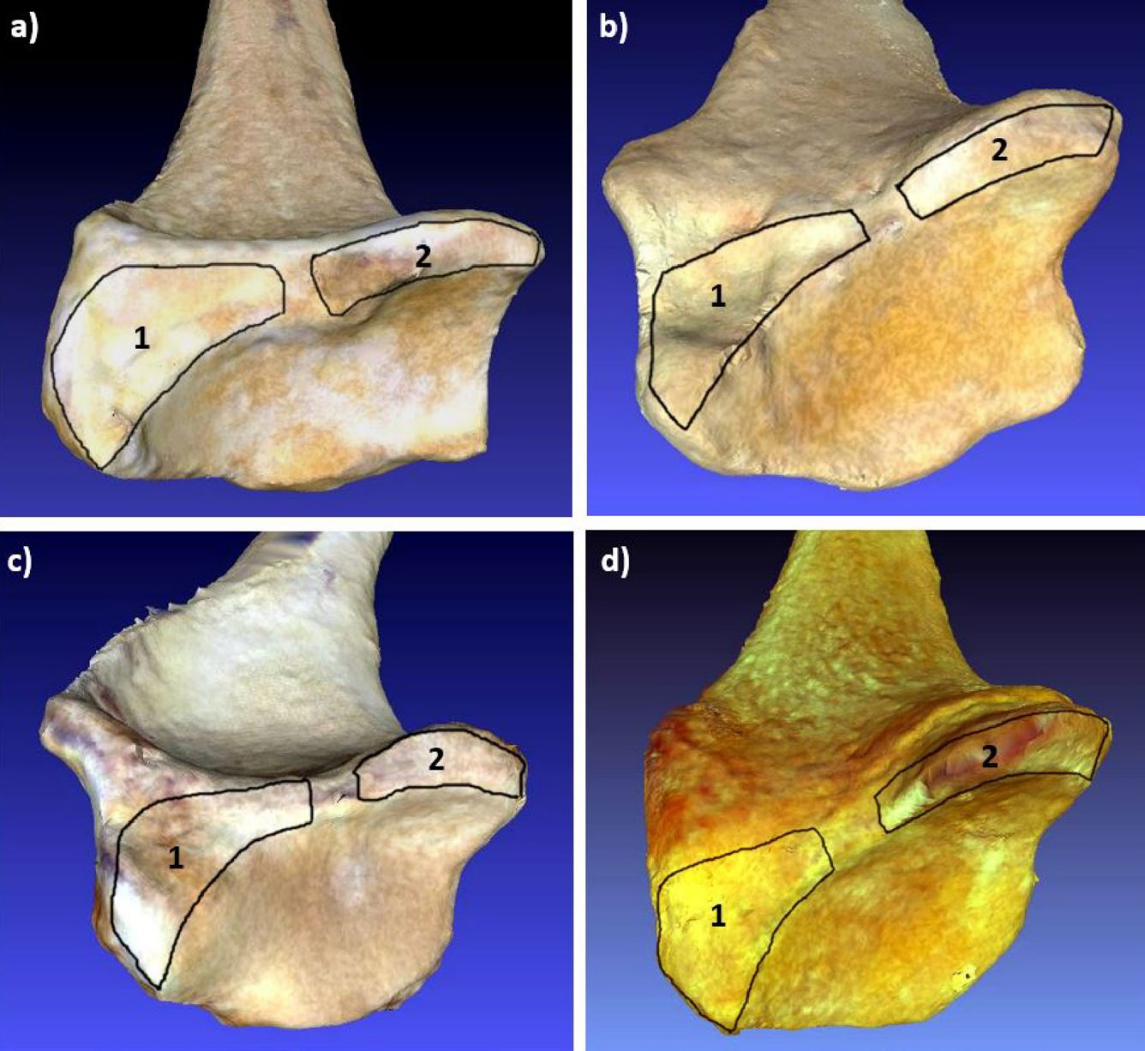
3D scans of the distal radial epiphysis in the four hominoid primate species analyzed. (a) Homo sapiens; (b) Pongo pygmaeus; (c) Pan troglodytes; (d) Gorilla gorilla. 1 = Common insertion area of the radioscaphocapitate and long radiolunate ligaments; 2 = Insertion area of the short radiolunate ligament.

We applied the set of landmarks proposed by Casado^29^ to capture the morphology of the two insertion areas of the three ligaments that comprise the palmar radiocarpal complex in the distal radial epiphysis (Fig. 1). Ten Type II and III landmarks were used: L1–L4 to represent the insertion site of the short radiolunate ligament (SRL) and L5–L10 for the common insertion area of the radioscaphocapitate (RSC) and long radiolunate (LRL) ligaments (Fig. 2). This protocol has been validated in previous work, demonstrating low intra- and inter-observer error and high repeatability^44^. Landmark coordinates were then exported into the MorphoJ statistical software package^45^ for geometric morphometric analyses.

A Generalized Procrustes Analysis (GPA) was performed to eliminate variation due to size, position, and orientation, minimizing the sum of squared distances between equivalent landmarks^46–48^. This step produces Procrustes coordinates, which were used for multivariate statistical analyses^48,49^. Principal Components Analysis (PCA) was applied to reduce multidimensional variation into a smaller number of components (eigenvectors) summarizing the major patterns of shape variation between groups^45,47,48^.

Canonical Variate Analysis (CVA) was then conducted to maximize shape differences among predefined groups^48^, and shape changes along canonical axes were visualized as landmark displacements relative to the mean shape. Group classification accuracy was evaluated using Linear Discriminant Analysis (LDA) with Fisher’s classification rule and a leave-one-out, jackknife cross-validation procedure to compute post hoc classification probabilities^45^.

Finally, a Multivariate Regression Analysis (MRA) was performed, with Procrustes coordinates (shape) as the dependent variable and centroid size (CS), indicative of distal radius size, as the independent variable, to test for potential allometric effects. This analysis used a permutation test with 1,000 randomizations^45–48^.

### Ethical note

The research complied with protocols approved by the Institutional Animal Care and Use Committee of the University of Barcelona (IRB00003099) and adhered to the legal requirements of Spain.

## Results

**Fig. 3:**
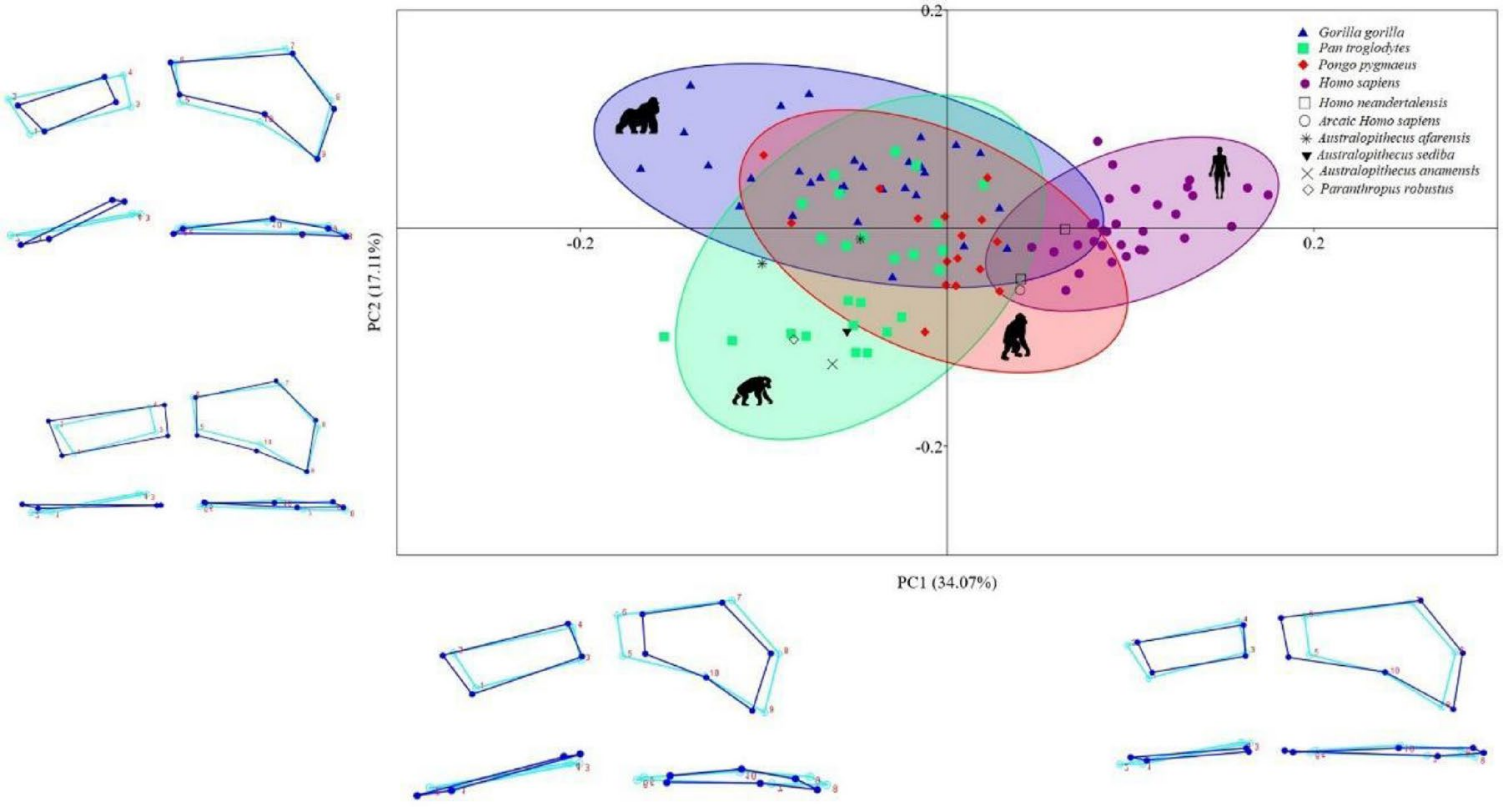
Scatter plot of PC1 versus PC2 derived from the PCA of the 3D GM analysis. 95% equal frequency ellipses of the groups are depicted. Dark blue wireframes show the extreme shape of each PC in a palmar view (upper panel) and a distal view (lower panel). Light blue wireframes show the mean shape (coordinates 0.0). *3D GM = three-dimensional geometric morphometrics; PCA = principal components analysis*.

The PCA identified 23 principal components (PC), the first five of which accounted for 73.86% of the variation in the shape of the two ligament insertion sites: PC1, 34.07%; PC2, 17.11%; PC3, 11.38%; PC4, 6.13%; PC5, 5.17%. The remaining PCs accounted for ≤ 5% each of the variation in shape. The scatterplot of PC1 vs. PC2 (Fig. 3) shows differences among the four species of hominoid primates, although there is a clear degree of overlap. *Homo sapiens*, *Homo neanderthalensis*, and archaic *Homo sapiens* are mainly located in the positive PC1 values, while the African hominoid primates (the chimpanzees and gorillas), *Australopithecus afarensis*, *Paranthropus robustus*, *Australopithecus anamensis* and *Australopithecus sediba* are mainly located in the negative values. *Pongo pygmaeus* is located in an intermediate position between the positive and negative values.

Specimens with positive PC1 values were characterized by a relatively large insertion site of the RSC and LRL ligaments and a relatively smaller insertion site of the SRL ligament, with a palmar orientation. In contrast, specimens with negative PC1 values had a relatively smaller insertion site of the RSC and LRL ligaments and a larger, more ulnopalmarly oriented SRL ligament insertion site. PC2 was able to differentiate between the knuckle-walkers, with *Gorilla gorilla* having mainly positive values, while *Pan troglodytes* had mainly negative values. In specimens with positive PC2 values, both insertion sites were relatively smaller and the SRL ligament insertion site had an ulnopalmar orientation. In specimens with negative PC2 values, both insertion sites were larger and the SRL ligament had a palmar orientation. PC3 values showed no differences among the four species.

**Fig. 4:**
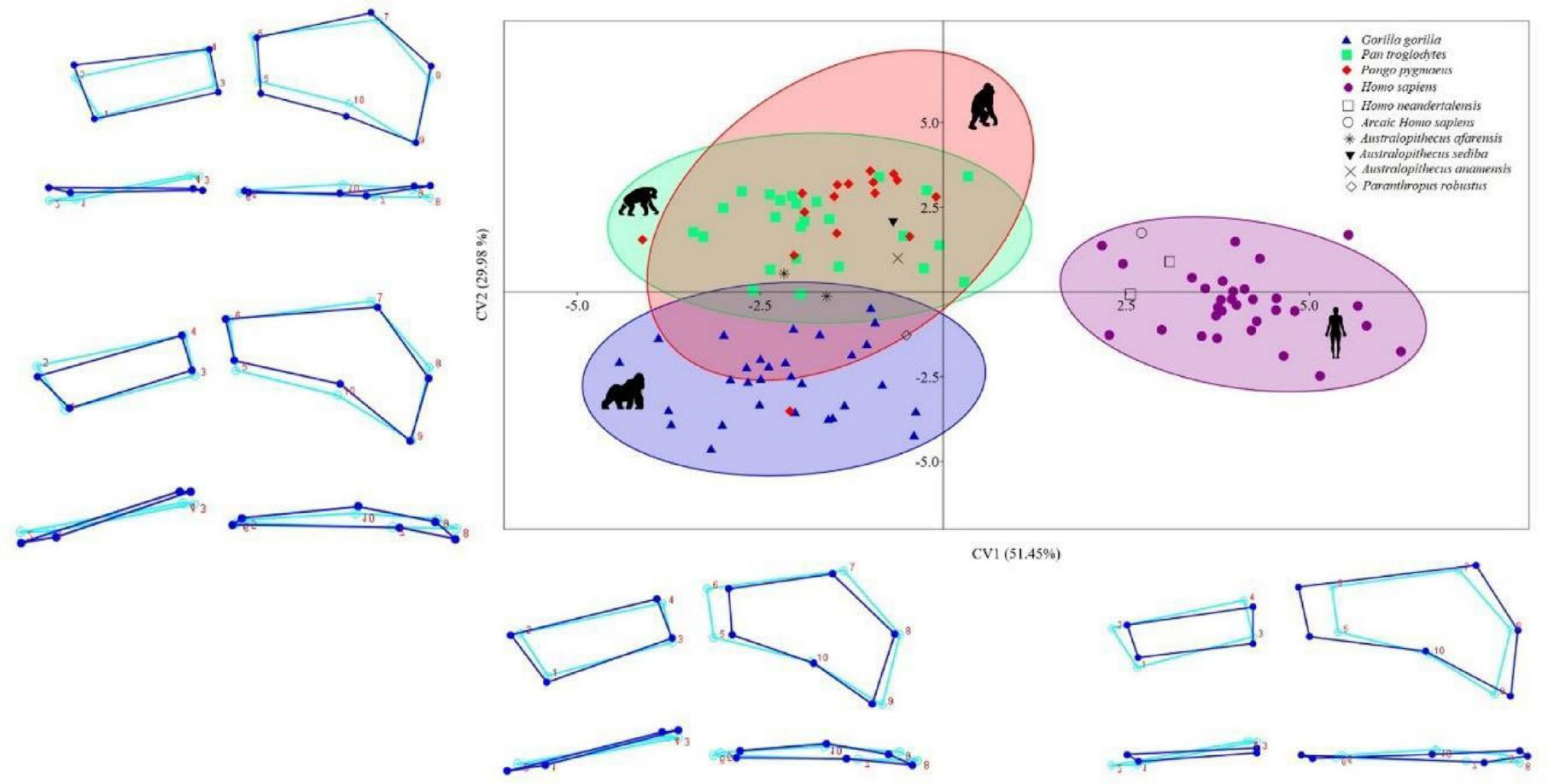
Scatter plot of CV1 versus CV2 derived from the CVA of the 3D GM analysis. 95% equal frequency ellipses of the groups are depicted. Dark blue wireframes show the extreme shape of each CV in a palmar view (upper panel) and a distal view (lower panel). Light blue wireframes show the mean shape (coordinates 0.0). *3D GM = three-dimensional geometric morphometrics; CVA = canonical variate analysis*.

The CVA (Fig. 4) identified nine canonical variates (CV), the first three of which accounted for 89.79% of the difference in shape of the ligament insertion sites: CV1, 51.45%; CV2, 26.98%; CV3, 11.36%. The remaining CVs accounted for ≤ 5% each of the variation in shape. *Homo sapiens*, *Homo neanderthalensis*, and archaic *Homo sapiens* are mainly located in the positive CV1 values, while the non-human hominoid primates, *Australopithecus afarensis*, *Paranthropus robustus*, *Australopithecus anamensis* and *Australopithecus sediba* are mainly located in the negative values. The CV2 was able to differentiate between the more arboreal hominoid primates (the chimpanzees and orangutans), which had mainly positive values, and the more knuckle-walkers (gorillas), which had mainly negative values. *Australopithecus afarensis*, *Australopithecus sediba* and *Australopithecus anamensis* had mainly positive CV2 values, while *Paranthropus robustus* had mainly negative values. The morphological characteristics of the ligament insertion sites according to CV1 and CV2 values were similar to those described for the PC1 and PC2 values.

Permutation tests run over Mahalanobis and Procrustes distances showed a good consistency between CVA (Table 2) and LDA (Table 3) values. Both CVA and LDA values for *Australopithecus afarensis* were significantly different from those of *Homo sapiens*, *Gorilla gorilla* and *Pongo pygmaeus*, but not from *Pan troglodytes*. Both CVA and LDA values for *Australopithecus anamensis* were significantly different from those of *Homo sapiens* and *Gorilla gorilla*, while CVA – but not LDA – values for *Australopithecus anamensis* were significantly different from those of *Pongo pygmaeus*. Neither CVA nor LDA values for *Australopithecus anamensis* were different from those of *Pan troglodytes*. *Australopithecus sediba* also had significantly different CVA and LDA values from those of *Homo sapiens* and *Gorilla gorilla*, but not from those of *Pan troglodytes* or *Pongo pygmaeus*. *Paranthropus robustus* had significantly different CVA values from those of *Homo sapiens*, *Pan troglodytes*, *Gorilla gorilla* and *Pongo pygmaeus*, while there were no significant differences in LDA values between *Paranthropus robustus* and *Pan troglodytes* or *Pongo pygmaeus*. Both CVA and LDA values were significantly different between *Homo neanderthalensis* and *Homo sapiens*, *Pan troglodytes*, *Gorilla gorilla* and *Pongo pygmaeus*. Finally, archaic *Homo sapiens* had significantly different CVA values from those of *Homo sapiens*, *Pan troglodytes* and *Gorilla gorilla*, but not from those of *Pongo pygmaeus*, and significantly different LDA values from those of *Homo sapiens* and *Gorilla gorilla*, but not from those of *Pan troglodytes* and *Pongo pygmaeus* (Tables 2 and 3).

**Table 2:** CVA Mahalanobis and procrustes (in italics) distances between groups with p-values based on 1000 permutations.

	*H. sapiens*	*P. troglodytes*	*G. gorilla*	*P. pygmaeus*
*A. afarensis*	7.33 (*p* = 0.001) *0.19* (*p* = 0.001)	4.42 (*p* = 0.09) *0.07* (*p* = 0.69)	5.10 (*p* = 0.005) *0.11* (*p* = 0.05)	5.75 (*p* = 0.005) *0.11* (*p* = 0.018)
*A. anamensis*	6.94 (*p* = 0.01) *0.22* (*p* = 0.007)	5.01 (*p* = 0.64) *0.13* (*p* = 0.29)	6.25 (*p* = 0.09) *0.18* (*p* = 0.01)	6.04 (*p* = 0.29) *0.17* (*p* = 0.04)
*A. sediba*	8.07 (*p* = 0.007) *0.19* (*p* < 0.001)	5.65 (*p* = 0.34) *0.09* (*p* = 0.82)	7.73 (*p* = 0.03) *0.16* (*p* = 0.01)	7.32 (*p* = 0.10) *0.14* (*p* = 0.22)
*P. robustus*	8.99 (*p* = 0.003) *0.23* (*p* = 0.006)	7.92 (*p* = 0.03) *0.13* (*p* = 0.21)	7.98 (*p* = 0.03) *0.18* (*p* = 0.01)	9.67 (*p* = 0.02) *0.08* (*p* = 0.02)
*H. neanderthalensis*	5.42 (*p* = 0.002) *0.11* (*p* = 0.001)	6.72 (*p* < 0.001) *0.13* (*p* = 0.03)	7.69 (*p* = 0.002) *0.17* (*p* = 0.002)	7.74 (*p* = 0.004) *0.13* (*p* = 0.006)
*H. sapiens archaic*	6.70 (*p* = 0.001) *0.14* (*p* = 0.004)	7.30 (*p* = 0.03) *0.14* (*p* = 0.21)	8.84 (*p* = 0.03) *0.19* (*p* = 0.01)	8.15 (*p* = 0.06) *0.14* (*p* = 0.25)

**Table 3:** LDA Mahalanobis and procrustes (in italics) distances between groups with P-values based on 1000 permutations.

	*H. sapiens*	*P. troglodytes*	*G. gorilla*	*P. pygmaeus*
*A. afarensis*	12.37 (*p* = 0.03) *0.18* (*p* < 0.001)	20.34 (*p* = 0.11) *0.07* (*p* = 0.68)	8.89 (*p* = 0.19) *0.11* (*p* = 0.05)	7.80 (*p* = 0.04) *0.11* (*p* = 0.02)
*A. anamensis*	14.78 (*p* = 0.09) *0.21* (*p* = 0.003)	14.02 (*p* = 0.71) *0.13* (*p* = 0.29)	11.91 (*p* = 0.27) *0.18* (*p* = 0.02)	6.09 (*p* = 0.31) *0.16* (*p* = 0.06)
*A. sediba*	19.19 (*p* = 0.002) *0.19* (*p* = 0.001)	22.26 (*p* = 0.4) *0.09* (*p* = 0.8)	12.11 (*p* = 0.20) *0.16* (*p* = 0.01)	6.90 (*p* = 0.35) *0.22* (*p* = 0.22)
*P. robustus*	24.50 (*p* = 0.03) *0.23* (*p* = 0.001)	31.12 (*p* = 0.20) *0.14* (*p* = 0.24)	14.81 (*p* = 0.07) *0.17* (*p* = 0.02)	7.78 (*p* = 0.9) *0.19* (*p* = 0.07)
*H. neanderthalensis*	18.53 (*p* = 0.001) *0.10* (*p* = 0.003)	16.62 (*p* = 0.20) *0.13* (*p* = 0.04)	10.49 (*p* = 0.06) *0.17* (*p* < 0.0001)	7.74 (*p* = 0.07) *0.12* (*p* = 0.007)
*H. sapiens archaic*	25.38 (*p* = 0.001) *0.14* (*p* = 0.005)	20.29 (*p* = 0.49) *0.14* (*p* = 0.22)	13.81 (*p* = 0.14) *0.19* (*p* = 0.03)	9.42 (*p* = 0.18) *0.14* (*p* = 0.23)

The post-hoc classification results from the discriminant functions and the leave-one-out cross-validation showed variations in the percentages of correct classification across different species (Table 4). The initial classification using the discriminant functions was 100% in all cases, indicating a perfect separation of the species. However, after cross-validation, the percentages decreased, with the exception of *Paranthropus robustus* vs. *Homo sapiens*. This reduction in accuracy following cross-validation suggests that, while the discriminant functions appropriately separate the groups, the robustness of the model varies between species.

**Table 4:** Percentages of correct *post-hoc* classification from the discriminant functions and after leave-one out cross-validation AAf = *Australopithecus afarensis*; AAn = *Australopithecus anamensis*; AS = *Australopithecus sediba*; PR = *Paranthropus robustus*; HN = *Homo neanderthalensis*; AHS = archaic *Homo sapiens*; HS = *Homo sapiens*; PT = *Pan troglodytes*; GG = *Gorilla gorilla*; PP = *Pongo pygmaeus*.

Comparison	Discriminant function (%)	Cross-Validation (%)	Decrease in correct classification (%)
*AAf vs. HS*	100	71.8	28.2
*AAf vs. PT*	100	68	32
*AAf vs. GG*	100	93.6	6.5
*AAf vs. PP*	100	68.3	31.7
*AAn vs. HS*	100	75	25
*AAn vs. PT*	100	65	35
*AAn vs. GG*	100	75	25
*AAn vs. PP*	100	65	35
*AS vs. HS*	100	75	25
*AS vs. PT*	100	65	35
*AS vs. GG*	100	75	25
*AS vs. PP*	100	65	35
*PR vs. HS*	100	100	0
*PR vs. PT*	100	84	16
*PR vs. GG*	100	90.3	9.7
*PR vs. PP*	100	65	35
*HN vs. HS*	100	71.8	28.2
*HN vs. PT*	100	65	35
*HN vs. GG*	100	68.6	31.5
*HN vs. PP*	100	71.7	28.3
*AHS vs. HS*	100	96.9	3.1
AHS vs. PT	100	96	4
*AHS vs. GG*	100	75	25
*AHS vs. PP*	100	65	35

The MRA of shape over CS was significant (*P* = 0.01), but only 2.11% of the variation in shape of the insertion sites could be attributed to size.

## Discussion

Our study of the ligament insertion sites in the distal radial epiphysis of different species of extant and extinct hominoid primates has revealed several morphological variations that reinforce current hypotheses about the evolution of locomotor behavior and manipulative skills in these primates. By using techniques of morphological analysis like PCA and CVA, we have been able to compare specific adaptations in the different species and to identify significant evolutionary patterns. Our results are consistent with traditional classifications of fossil hominins, which distinguish between species that use only bipedal locomotion and those that combine bipedalism with arboreal-type locomotion, a division that reflects the different evolutionary trajectories of each species^50^.

Importantly, our approach goes beyond traditional osteological comparisons by incorporating enthesis-based analyses, which provide valuable functional information about soft tissue adaptation and loading regimes. In particular, the use of three-dimensional geometric morphometrics (3D GM) to examine ligament insertion sites allows us to infer differences in habitual mechanical loading that are not necessarily visible from bone morphology alone. This aligns with recent research employing the VERA method (Validated Entheses-based Reconstruction of Activity), which has demonstrated that the shape and position of muscle and ligament entheses can reliably reflect habitual activity patterns in both extant and fossil hominins^26,32^.

In this context, our study explicitly evaluates the hypothesis that the morphology of ligament entheses integrates both evolutionary constraints and functional plasticity, rather than treating these factors as separate. We propose that these insertion sites act as complex anatomical markers that encode phylogenetic history while reflecting mechanical demands throughout life, making them highly informative structures for reconstructing locomotor strategies and manipulative behaviors in extinct hominins. This interpretation is supported by previous research showing that the morphology of palmar radiocarpal ligament insertions varies predictably across great apes according to locomotor patterns^21,22,29^ and that these entheses exhibit measurable plastic responses to mechanical loading, as demonstrated in captive primates^22^.

Interestingly, we found that *Homo neanderthalensis* and archaic *Homo sapiens* cluster with *Homo sapiens* in both the PCA (Fig. 3) and the CVA (Fig. 4), suggesting a shared morphological adaptation related to the use of bipedalism, the absence of arboreal-type locomotion, and the specialization of the upper limbs in manipulative tasks^29^, all of which are distinctive features in the evolution of the genus *Homo*^51^. This similarity was expected, functioning as a “sanity check” of the methodology, but our results also reveal a more nuanced pattern. Both *Homo neanderthalensis* and archaic *Homo sapiens* exhibit a relatively larger and more ulnopalmar-oriented SRL insertion site compared to modern humans (Tables 2 and 3; Figs. 2 and 3). This morphological signal suggests that these fossil populations retained robust wrist mechanics, potentially linked to high manual loading demands such as forceful gripping, tool production, and environmental manipulation. The SRL plays a crucial role in stabilizing the wrist during extension and ulnar deviation^52,53^, the main functional movements of the hand. Thus, the larger and ulnopalmar-oriented SRL insertion site observed in fossil humans supports the idea that *Homo neanderthalensis* and archaic *Homo sapiens* were adapted to perform manual tasks requiring greater physical effort than those typically undertaken by modern humans^54^. In contrast, modern *Homo sapiens* shows a relatively smaller SRL insertion site with a more palmar orientation, which corresponds to a wrist optimized for precise manipulation, reflecting an evolutionary trend toward activities demanding fine motor control and advanced tool use^55,56^. These findings add functional depth to their phylogenetic proximity and indicate that archaic humans may have maintained greater ligamentous stabilization than contemporary *Homo sapiens*.

This shift from robust to refined manipulation is further supported by recent entheseal studies showing that modern humans have distinct patterns of muscle recruitment compared to other primates, reflecting habitual use of precision grips rather than power grips^35^. In this context, our findings at the level of the SRL ligament insertion sites converge with broader trends observed in thumb entheses and carpal morphology.

In this regard, we recognize that modern post-industrial osteological collections do not necessarily represent the ancestral condition of *Homo sapiens*^57^. Sedentary lifestyles, dietary changes associated with industrialization, and intensive use of technology have influenced skeletal morphology, particularly in long bones and the spine. However, this bias has limited impact on the wrist, whose biomechanical role is highly conserved and subjected to a stable load pattern that includes axial compression forces transmitting approximately 80% of loads through the radius and 20% through the ulna via the triangular fibrocartilage complex, shear forces generated during radial and ulnar deviation, peak flexion-extension moments at 60–80° typical of falls, torsional stresses derived from pronation-supination movements, and repetitive micro-loads of low magnitude that shape the ligamentous and osseous architecture over a lifetime. This anatomical specialization for fine manipulation restricts the effect of lifestyle changes on carpal morphology, making this bias negligible in the context of our study^53^.

The morphology of the ligament insertion sites in the fossil hominins *Australopithecus afarensis*, *Australopithecus anamensis* and *Australopithecus sediba* were similar to that of non-human hominoid primates, as indicated by their position in the negative values of PC1 (Fig. 3) and CV1 (Fig. 4). In addition, the position of these three species in the negative values of PC2 (Fig. 3) and the positive values of CV2 (Fig. 4) indicates that they can be grouped with chimpanzees and orangutans – non-human hominoid primates with a greater use of arboreal locomotion^58^. This result is in line with conclusions reached by other investigators, who suggest that the genus *Australopithecus* combined bipedalism with a high degree of arboreal locomotion^2,59–62^. The main morphological adaptation to arboreal locomotion in the genera *Pan*, *Pongo* and *Australopithecus* is a greater relative size and an ulnopalmar orientation of the SRL insertion site (Figs. 2 and 3), which is mainly related to vertical climbing, where a large ulnar deviation of the hand translates into an increase in the loads affecting the radiolunate joint^63,64^. These greater loads can be compensated by a larger size of the SRL and by an ulnopalmar orientation of its insertion site^29^. The morphological similarities of the ligament insertion sites in the three *Australopithecus* species and the *Pan* and *Pongo* genera is in line with the results of the permutation tests carried out with the CVA and the LDA (Tables 2 and 3), where neither *Australopithecus afarensis* nor *Australopithecus anamensis* presented significant differences with *Pan troglodytes* while *Australopithecus sediba* showed no differences with *Pan troglodytes* or *Pongo pygmaeus* – perhaps due to their greater degree of arboreal locomotion. We also observed a high percentage of error in the post hoc classification when comparing the three species of *Australopithecus* with *Pan troglodytes* and *Pongo pygmaeus* (Table 4), which indicates a high degree of morphological similarity in their ligament insertion sites.

The morphology of the ligament insertion site of the *Paranthropus robustus* specimen included in our study was similar to that of the three *Australopithecus* species but with some slight differences. Like *Australopithecus*, *Paranthropus robustus* had negative PC1 and CV1 values (Figs. 2 and 3), which would indicate a morphology compatible with a combination of bipedalism and arboreal-type locomotion, as other authors have proposed for this genus^65,66^. However, unlike the three species of *Australopithecus*, *Paranthropus robustus* had negative CV2 values (Fig. 4), similar to those of gorillas, which may indicate that *Paranthropus robustus* had a higher degree of terrestrial locomotion than the genus *Australopithecus*, as has been suggested by other investigators^67^. It is also possible that some aspects of *Paranthropus* wrist morphology reflect convergent evolution toward terrestriality, rather than retention of ancestral traits. Further analyses with additional specimens are needed to clarify this pattern.

Taken together, our findings demonstrate that ligamentous entheses provide a powerful and underutilized window into past behavior. By applying 3D GM to the distal radial epiphysis, we contribute to the growing field of functional paleoanthropology that seeks to reconstruct the activity patterns of extinct species through biomechanical proxies. However, we also recognize that entheseal morphology is influenced by multiple factors, including age, sex, and pathology^23,25^, and thus should always be interpreted within a broader anatomical and ecological framework.

In summary, our findings reveal distinctive morphological characteristics of the ligament insertion sites on the distal radial epiphysis in modern humans, in non-human hominoid primates and in fossil hominins, highlighting the influence of locomotor behavior on the evolution of wrist anatomy. The divergent evolutionary trajectories observed in the PCA and CVA analyses clearly reflect morphological differences between bipedal individuals, arboreal primates, and hominins that combine bipedalism with varying degrees of arboreal locomotion. *Homo sapiens*, *Homo neanderthalensis* and archaic *Homo sapiens* show clear adaptations towards the use of their upper limbs for precise manipulation, in conjunction with their bipedal locomotion, while representatives of the genera *Australopithecus* and *Paranthropus* conserve the fundamental traits of arboreal locomotion, underscoring the evolutionary diversity within hominins. Whenever possible, it is clearly important to consider both bone structures and soft tissue insertions when studying the evolution of locomotion in primates. A methodological approach that combines the study of soft tissues with the morphometric analysis of their insertion sites can offer a broader and more detailed perspective on the evolution of hominins in response to their environments and needs.

### Conclusions

The present study has allowed us to confirm the existence of diverse locomotor behaviors in fossil hominins based on the analysis of the ligament insertions on the distal radial epiphysis. Ligaments are a type of soft tissue rarely used in studies of hominin locomotion, and our methodology thus provides novel information on this issue. The robust findings of our 3D GM analysis indicate that both *Australopithecus* and *Paranthropus* had significant adaptations to arboreal locomotion. This is in line with findings of previous studies but our use of ligament insertion sites provides a new perspective that enriches existing conclusions and offers a more precise view of how these early hominins retained the ability to move effectively in arboreal environments. Additionally, we have observed significant differences between the three groups of *Homo* included in our study. Although *Homo neanderthalensis* and archaic *Homo sapiens* share manipulative ability with modern humans, there are differences in the degree of development of the wrist ligaments, suggesting that *Homo neanderthalensis* and archaic *Homo sapiens* were better adapted to an environment that required greater physical strength, while *Homo sapiens* evolved a greater manual dexterity. This finding highlights a divergent evolutionary trajectory of the manipulative function of the wrist, especially as it relates to the use of tools and other activities requiring manual precision.

Our 3D GM analysis of the ligament insertion sites not only confirms previous hypotheses on the locomotion of *Australopithecus* and *Paranthropus* but also introduces an innovative methodology that complements traditional studies focusing on bone morphology. The ability to infer locomotor and manipulative patterns from 3D GM findings on these insertion sites suggests that we can expand the scope of application of this method to other evolutionary studies of both fossil and extant species. Our results demonstrate the value of this technique in the reconstruction of locomotor behavior, making it a valuable tool for future studies in the fields of paleontology and human evolution.

### Limitations of the study

Despite the methodological robustness of the present work, several limitations should be acknowledged. First, the use of modern post-industrial human osteological collections may introduce biases related to lifestyle, activity patterns, and health conditions that do not necessarily reflect the variability of ancestral Homo sapiens populations. In addition, the limited number of fossil specimens analyzed constrains the generalizability of the results and may increase the influence of intraspecific variability. Moreover, the morphology of ligamentous entheses can be influenced by factors not controlled in this study, such as age, sex, physical activity, or pathological conditions, which requires cautious interpretation of the findings. Finally, although three-dimensional geometric morphometrics provides a powerful tool for characterizing shape variation, the inference of locomotor and manipulative patterns from these structures necessarily relies on indirect correlations; therefore, future research should integrate this approach with complementary lines of evidence, including anatomical, biomechanical, and archaeological data.

## Data Availability

The fossil specimens and digital models analyzed in this study belong to institutional collections subject to strict access regulations. Our research team holds permission for their use but does not have distribution rights over either the original fossils or their 3D replicas. For legal and intellectual property reasons, the 3D files cannot be made publicly available. Researchers interested in accessing these materials must submit requests directly to the corresponding institutions.
